# Rapid Recovery of Postoperative Pulmonary Function in Patients With Lung Cancer and Influencing Factors

**DOI:** 10.3389/fonc.2022.927108

**Published:** 2022-07-11

**Authors:** Yang Fuzhi, Tang Dongfang, Fu Wentao, Wang Jing, Wu Yingting, Mo Nianping, Gao Wen, Shen Xiaoyong

**Affiliations:** Department of Thoracic Surgery, Huadong Hospital Affiliated to Fudan University, Shanghai, China

**Keywords:** lung cancer, pulmonary function, pulmonary rehabilitation, respiratory training, surgery

## Abstract

Among malignant tumors, lung cancer has the highest morbidity and mortality worldwide. Surgery is the first-line treatment for early-stage lung cancers, and has gradually advanced from conventional open-chest surgery to video-assisted thoracic surgery (VATS). Additionally, increasingly smaller surgical incisions and less surgical trauma have resulted in reduced pulmonary function damage. Previous studies have found that the level of pulmonary function loss and recovery is significantly correlated with postoperative complications and the quality of life. Thus, an accurate assessment of the preoperative pulmonary function and effective rehabilitation of postoperative pulmonary function are highly important for patients undergoing lung surgery. In addition, pulmonary function assessment after pulmonary rehabilitation serves as an objective indicator of the postoperative pulmonary rehabilitation status and is crucial to facilitating pulmonary function recovery. Furthermore, a complete preoperative assessment and effective rehabilitation are especially critical in elderly patients with pulmonary tumors, poor basic physiological functions, comorbid lung diseases, and other underlying diseases. In this review, we summarize the clinical significance of pulmonary function assessment in patients undergoing lung cancer surgery, postoperative changes in pulmonary function, effective pulmonary function rehabilitation, and the influencing factors of pulmonary function rehabilitation.

## Introduction

Among malignant tumors, lung cancer has the highest morbidity and mortality in China and throughout the world (1). Surgery is the first-line treatment for early-stage lung cancers, and the preoperative pulmonary function status and surgical methods are important factors affecting the prognosis and quality of life (2). With an inability to regenerate new tissues from residual pulmonary tissues, the surgical excision of functional tissues and reconstruction of residual thoracic structures results in reduced postoperative pulmonary function (3). In particular, wide ranging complications resulting from reduced respiratory function caused by pneumonectomy are major reasons for poor postoperative quality of life (4). Hence, an accurate assessment of preoperative pulmonary function is crucial in patients undergoing pneumonectomy (5). Here, we summarize the clinical significance of pulmonary function assessment in patients undergoing lung cancer surgery, postoperative changes in pulmonary function, effective pulmonary function rehabilitation, and the influencing factors of pulmonary function rehabilitation.

## Clinical Significance of Pulmonary Function Measurement in Patients Undergoing Lung Cancer Surgery

### Pre-Operative Pulmonary Function Assessment

Among pulmonary function indices, the forced expiratory volume in the 1st second (FEV1) and the diffusing capacity of the lung for carbon monoxide (DLCO) show the best correlations with postoperative morbidity and mortality rates (6). The predicted post-operative FEV1 (ppoFEV1) and forced vital capacity (ppoFVC) are highly correlated with the patient’s actual FEV1 and forced vital capacity (FVC) (r= 0.867, 0.832, respectively) (7). Hence, the preoperative measurement of pulmonary function and calculation of predicted postoperative values can be instrumental in effectively evaluating surgical feasibility and predicting postoperative pulmonary function recovery and pulmonary complications. There are several ways to predict postoperative lung function, including counting residual lung lobes, perfusion scintillation to calculate residual lung function, or imaging to calculate the area (8). The American College of Chest Physician’s evidence-based clinical practice guidelines (3rd edition) recommend the use of perfusion to calculate and predict lung function, using the following formulas:

Pneumonectomy: ppoFEV1 = preoperative FEV1 × (1 − fraction of total perfusion for the resected lung)

Lobectomy: ppoFEV1 = preoperative FEV1 × (1 − number of functional or unobstructed lung segments to be removed/total number of functional segments)

The same equations can be used to estimate the ppoDLCO. Both a ppoFEV1 and ppoDLCO >60% indicate a low risk for anatomic pneumonectomy (6).

A study by Zhang et al., comprising 805 patients undergoing pulmonary surgery, suggested that two indices, ppoFEV1% and ppoDLCO%, can be used to evaluate surgical feasibility and predict the risk of pulmonary complications, regardless of whether the surgery is open or minimally invasive (9). Furthermore, Khullar et al. indicated that DLCO might be a better predictor of the postoperative quality of life than age (10).

In summary, pre-operative pulmonary function assessment has significant value in predicting surgical feasibility, postoperative complications, and the quality of life. Thus, pulmonary function should be properly assessed preoperatively.

### Postoperative Pulmonary Function Testing

Patients undergoing lung resection surgery usually experience pulmonary function decline to various degrees after surgery. Generally, pulmonary function gradually recovers within 6-12 months postoperatively, depending on the range of the surgical excision and the intensity of postoperative rehabilitation exercise. Pulmonary function testing during hospitalization is conducive to the early detection of pulmonary infections. Gregor et al. performed postoperative pulmonary function testing in patients with lung cancer and found that the FEV1 decline at 4 days postoperatively was more notable in patients with pneumonia than in patients without pneumonia (43.2% VS 32,2%) (11). Hence, regular postoperative pulmonary function testing, combined with other diagnostic assessments such as laboratory testing, radiology, and breathing frequency, could help detect pneumonia. Furthermore, for patients undergoing rehabilitation exercise, pulmonary function assessment can be helpful in objectively evaluating the efficacy of the rehabilitation exercise. In addition, some researchers have found that the decline in postoperative pulmonary function is negatively correlated with the postoperative quality of life (12). However, a study by Ozturk et al. found no significant correlations between pulmonary function test parameters (FEV1, FVC, and FEV1/FVC) and the quality of life (13). Therefore, other cardiopulmonary function indices, such as the six-minute walk test (6MWT), might serve as better indicators of the postoperative quality of life (14).

## Postoperative Changes in Pulmonary Function in Patients With Lung Cancer

Lung surgery exerts notable short-term and long-term impacts on the postoperative pulmonary function of patients with lung cancer. There are various causes of pulmonary function impairment after pneumonectomy, including the resection of lung tissues and changes in the mechanical structure of the chest wall induced by surgical incisions (15). Conventional open-chest surgery results in a 10%–40% postoperative decrease in pulmonary function (16, 17). Postoperative pulmonary function assessment in a prospective study, comprising 238 patients undergoing muscle-sparing lateral thoracotomy lobectomy, found that the FEV1 and DLCO values at 3 months postoperatively were only 84% and 88.5% of preoperative values, respectively (16). Shiono et al. revealed a significant decrease in pulmonary function at two weeks postoperatively and a significant decrease in pulmonary function and oxygenation at 6 months postoperatively (2). The adoption of VATS within the division of thoracic surgery aids in sparing more chest wall muscle, reducing surgical trauma and pulmonary function loss. A retrospective study by Shibazaki et al., comprising 104 patients who underwent VATS lobectomy reported that the mean FEV1 at 3, 6, and 12 months postoperatively was 85.78%, 87.93%, and 89.22% of preoperative values, respectively (18). Nezu et al. reported a significant decrease in patients’ pulmonary function at 3 months after pneumonectomy, and improvement in pulmonary function at 6 months, but with a failure to return to preoperative levels (19). In a prospective study of patients undergoing unilateral VATS lobe resection, Yokoba et al. found that FVC and FEV1 values at 3-12 months postoperatively were lower than preoperative vales (20).

In brief, a long-term postoperative decline in pulmonary function is inevitable, regardless of whether the surgery is open-chest or VATS. Although pulmonary function gradually improves after surgery, the overall level is still lower than the preoperative level, which affects the postoperative quality of life to varying degrees.

### Factors That Affect Postoperative Pulmonary Function Recovery

Pulmonary function recovery after lung cancer surgery is affected by various factors. The choice of surgical method and the range of lung tissue excision are major factors of pulmonary function decline (21). Smoking history and body mass index (BMI) are also important factors affecting postoperative FEV1 (18). Moreover, concomitant pulmonary diseases (4) and effective postoperative rehabilitation (22) can affect pulmonary function recovery.

### General Pre-Operative Condition Factors

General pre-operative condition factors, including age (23), BMI (24), smoking history, concomitant lung diseases, and other underlying diseases, etc.: Pulmonary functions begin to decline at the age of 35 years, with a mean FEV1 decline of approximately 30 mL/year and a mean FVC decline of approximately 20 mL/year (25). Mori et al. found that younger age was related to greater postoperative pulmonary function recovery (26). Elderly patients usually have comparatively poor pulmonary function; thus, special attention should be paid to the preoperative assessment of pulmonary function and postoperative recovery (27).

Compared to normal weight and obesity, underweight status is associated with decreased pulmonary function (24). Good preoperative nutritional status can facilitate postoperative pulmonary function recovery and reduce complications (28). For patients with poor nutritional status, perioperative nutritional support should be strengthened to reduce postoperative complications and facilitate pulmonary function recovery. Central obesity is associated with pulmonary function decline in the Chinese elderly population, with better pulmonary function in patients with moderate obesity (29); proper diet control and postoperative rehabilitation exercise can facilitate the improvement in pulmonary function in such patients.

Long-term smoking is a risk factor for pulmonary complications after pneumonectomy, and smoking cessation can effectively lower the incidence of pulmonary complications (30, 31). However, the level of impact that smoking has on postoperative pulmonary function recovery currently remains unclear and needs to be confirmed by further observations and studies.

The process of pulmonary function recovery after lung cancer surgery is accompanied by various influencing factors, including pulmonary atelectasis, pleural effusion, and postoperative chest pain. After lung cancer surgery, early postoperative rehabilitation and improvement in pulmonary function should be facilitated by the aggressive administration of expectorants, proper use of analgesics, tapping on the back in a prone position (to encourage coughing, facilitate postoperative sputum excretion, and promote re-expansion of collapsed lung lobes), improvement in pulmonary ventilation and gas-exchange function, and aggressive treatment of complications, including pleural effusion and pulmonary infections.

### Choice of Surgical Method

Surgical trauma is an important factor influencing postoperative pulmonary function recovery. Posterolateral incisions are most widely used in conventional open-chest surgery. The incisions are relatively long and the latissimus dorsi muscle needs to be incised. Additionally, intraoperative rib spreading usually damages the ribs and intercostal nerves, which causes persistent postoperative pain and affects pulmonary function recovery. Liu et al. showed greater FVC decline and FVC% decline at 6 months and 2 years postoperatively with pneumonectomy and chest wall resection than with chest wall-sparing pneumonectomy (21). Harada et al. and Macke et al. reported significant pulmonary function decline in patients who underwent chest wall resection, and better pulmonary function recovery in patients receiving VATS lobectomy than in patients who underwent open lobectomy (especially in the early postoperative period); thus, pulmonary function recovery needs to be facilitated in the early period (32, 33). Lung surgery with VATS spares more chest wall muscle, cause less surgical trauma and less respiratory muscle injury, than conventional open-chest surgery, and hence are more conducive to postoperative pulmonary function recovery.

### The Range of Lung Tissue Excision

With the development of minimally invasive techniques, most lung cancer surgeries have been performed by VATS in recent years. The range of lung tissue excision varies depending on the size, amount, and location of the lesions, which are major factors affecting postoperative pulmonary function recovery. Mori et al. demonstrated that, in patients who underwent lung wedge resection, postoperative FVC decreased temporarily, but nearly recovered to the preoperative level after 12 months, whereas the postoperative FEV1 recovered gradually over the course of 12 months, but did not recover to the preoperative level (26). Furthermore, the number of resected lung segments is significantly and positively correlated with the FVC decline (32). FEV1 decline is greater in patients who underwent the resection of 3-5 segments than in patients who underwent the resection of fewer anatomical segments (1-2 segments), suggesting that the resection of less lung tissue results in less pulmonary function loss (33). Saito et al. and Keenan et al. found that decreases in FVC and FEV1 were notably smaller after the resection of pulmonary segments than after pulmonary lobectomy (34, 35). Since a smaller amount of lung tissue is resected, pulmonary functions are better preserved with pulmonary segmentectomy and lung wedge resection than with pulmonary lobectomy and the resection of multiple pulmonary segments. It is worth mentioning that sleeve lobectomy preserves more lung tissue and pulmonary function than pneumonectomy. In particular, for some patients with poor preoperative pulmonary function, sleeve resection could help preserve pulmonary function.

Therefore, when choosing the surgical method, surgeons should consider retaining normal lung tissues when possible, on the premise of complete tumor resection, in order to facilitate pulmonary function recovery in the early postoperative period and improve the quality of life.

### Comorbid Chronic Obstructive Pulmonary Disease

Changes in pulmonary function are associated with the location, volume, and severity of emphysema (20). Lung volume reduction surgery (LVRS) is a surgical intervention for patients with emphysema to improve lung function. LVRS is a potential option for patients with upper lobe emphysema and low exercise tolerance. The National Emphysema Treatment Trial showed that, compared with thoracotomy lung volume reduction, LVRS leads to better improvements in 6MWT distance, predictive FEV1%, quality of life, and dyspnea. Upper lobe–dominant emphysema and inhomogeneous emphysema showed better recovery of lung function than lower lobe–dominant emphysema and homogeneous emphysema (36). Comorbid COPD is an independent favorable factor for the preservation of FEV1 in the late postoperative phase following lung cancer surgery (4). The postoperative pulmonary function and recovery time in patients who undergo VATS pulmonary lobectomy vary depending on the pulmonary lobectomy itself and the presence of COPD (37). Wei et al. reported that the decline in postoperative FEV1 among patients with lung cancer and comorbid COPD ranged 5%–18.3%, indicating that pulmonary lobectomy did not cause further pulmonary function impairment in patients with lung cancer and comorbid COPD (38). Baldi et al. reported that patients with lung cancer and comorbid COPD exhibited a smaller decrease in pulmonary function during the late postoperative phase after pulmonary lobectomy than in patients without comorbid COPD (39). This phenomenon might be associated with reduced lung volume after pneumonectomy, alleviation of pulmonary hyperinflation, and changes in the mechanical structure of the chest wall. Further studies are required to confirm the specific mechanisms.

### Postoperative Pulmonary Function Compensation

Compensation for reduced pulmonary functions after pneumonectomy can help slow the pulmonary function decline caused by surgery. Physiological compensation after the resection of lung tissues is mainly achieved *via* two mechanisms: enhancement of the diffusing capacity of the residual lung and the generation of new pulmonary gas-exchange units (40). Fisher et al. demonstrated an increase in the mitotic activity of contralateral alveolar cells in rats after unilateral pneumonectomy, suggesting that functional compensation after pneumonectomy is primarily a compensation of the residual lung (41). This compensation is manifested, not by an overexpansion of the pre-existing alveolar septal tissues, but instead by an increase in functioning lung tissue (42). Ueda et al. showed that, despite the removal of more functional pulmonary parenchyma, postoperative pulmonary function after lower lobe resection was not worse than that after upper lobe resection because of greater postoperative compensation with lower lobe resection (5). Postoperative compensation after lower lobe resection is achieved by the expansion of both the contralateral lung and remaining ipsilateral lung. Hence, the postoperative decrease in total lung volume after lower lobe resection is smaller than that after upper lobe resection, even though more pulmonary parenchyma are excised in lower lobe resection (43).

### Preoperative and Postoperative Therapy

For patients with locally advanced lung cancer, neoadjuvant therapies, (including radiotherapy, chemotherapy, targeted therapy, and immunotherapy) can cause some damage to normal lung tissues while treating primary lesions, resulting in pulmonary function decline after treatment. Nomori et al. demonstrated pulmonary function decline in patients who underwent induction radiotherapy and chemotherapy; the pulmonary function decline primarily occurred on the affected side (44), which was probably the result of damage to the surrounding normal lung tissues caused by the radiotherapy. Radiation exposure in radiotherapy can lead to radiation pneumonitis in the early phase of treatment and pulmonary fibrosis in the late phase, which causes progressive dyspnea with decreased lung compliance, and, hence, lower FEV1 and DLCO (45). Shin et al. revealed a notable decrease in DLCO following neoadjuvant chemotherapy (46). Zhu et al. suggested that preoperative neoadjuvant immunotherapy significantly increased FEV1 and FEV1% while treating the primary tumor, enabling pathological remission, but also caused a decline in DLCO% (47). This might be due to damage to normal lung tissues caused by neoadjuvant therapies, resulting in reduced pulmonary gas-exchange function, despite an improvement in ventilation function induced by tumor volume reduction. At present, there remains a lack of studies regarding the impact of postoperative adjuvant therapies on the pulmonary function of patients with advanced lung cancer; thus, this topic requires further studies and discussions. For patients with locally advanced lung cancer, complications of chemotherapy and radiotherapy, including pneumonia and pulmonary fibrosis, should be prevented and treated; furthermore, aggressive rehabilitation treatment is conducive to pulmonary function improvement (45).

### Effective Rehabilitation Exercise

Poor exercise capacity (peak oxygen consumption <15 mL/kg/min) is the major determinant of postoperative morbidity and mortality after pneumonectomy (48). Effective pulmonary rehabilitation can improve cardiopulmonary function and exercise tolerance, and has positive clinical value in improving long-term survival (49–53). Cesario et al. showed notable improvement in FEV1 at 1 month postoperatively with in-hospital pulmonary rehabilitation than without pulmonary rehabilitation (2.32 Lt vs 1.79 Lt) (22). A randomized controlled trial by Zhou et al. demonstrated higher FEV1 at 28 days postoperatively in patients receiving pulmonary rehabilitation than in the control group (54). A study on patients receiving thoracoscopic lobectomy or segmental resection by Zou et al. found more notable improvement in pulmonary function at 3 months after hospital discharge in patients receiving pulmonary rehabilitation treatment (55). Furthermore, a retrospective analysis of patients who underwent VATS pneumonectomy by Choi et al. revealed better FEV1 preservation in the rehabilitation group than in the non-rehabilitation group (56). These results indicate that effective rehabilitation exercise can facilitate pulmonary function improvement.

However, unlike the above studies, a 20-week trial by Edvardsen et al. showed no difference in FEV1 between the rehabilitation group (who underwent high-intensity endurance and strength training) and the control group; only DLCO was higher in the rehabilitation group (57). Studies by Jonsson et al. and Cavalheri et al. suggested no difference in physical activity and pulmonary function at 8-12 weeks postoperatively between the rehabilitation and control groups (58, 59). This result might be associated with the low exercise intensity in the rehabilitation group. Furthermore, the intervention duration in these studies was short, at less than 3 months. There is currently no standardized procedure for pulmonary rehabilitation; different research institutions have adopted different postoperative rehabilitation programs, and hence might reach different conclusions. In addition, pulmonary function testing alone cannot fully reflect the cardiopulmonary function status. Pulmonary function testing combined with other exercise endurance assessments (such as the 6MWT and stair-climb test) might serve as a more accurate indicator of pulmonary rehabilitation efficacy. Previous studies have shown that pulmonary function recovers in about 6-12 months. In order to better improve the pulmonary function of patients, postoperative rehabilitation exercise should ideally last approximately 1 year. Furthermore, rehabilitation exercises should reach a certain intensity and be personalized to the patient’s individual tolerability. Patient compliance is an important factor affecting the execution of rehabilitation programs; thus, rehabilitation therapies should be individualized and tailored to the patient’s condition during the process of pulmonary rehabilitation, in order to achieve the best postoperative rehabilitation outcomes.

Clinical experience suggests that postoperative patients should perform proper breathing and aerobic exercises in the early postoperative phase, including deep breathing, pursed-lip breathing, jogging, swimming, stair climbing, etc. In addition, pulmonary function tests should be regularly performed; the exercise intensity can be gradually increased and maintained for 3-6 months, based on the pulmonary function recovery and improvement in respiratory symptoms. These measures can be instrumental in accelerating the postoperative pulmonary function recovery.

## Conclusions

With continued progress in minimally invasive concepts and techniques, VATS exerts an increasingly smaller impact on postoperative pulmonary function. Thus, there exists some controversy regarding the pulmonary function changes in patients undergoing lung cancer surgery; it was previously believed that lung cancer surgery can cause an absolute decline in postoperative pulmonary function, affecting postoperative complications and the quality of life. However, in recent years, some researchers have suggested that postoperative pulmonary function in patients with lung cancer can basically recover within 6-12 months after surgery despite a transient decline, without affecting the postoperative quality of life. Such studies mainly focused on patients receiving lung segmental and lung wedge resections, who experience a mild decline in postoperative pulmonary function. After prolonged recovery and compensation for pulmonary function loss, their pulmonary function basically recovers to the preoperative level, with little impact on the postoperative quality of life. In terms of postoperative pulmonary rehabilitation, most studies suggest that cardiopulmonary function and the quality of life can be improved to a certain extent after lung cancer surgery *via* effective pulmonary rehabilitation exercise. However, some studies suggest that pulmonary rehabilitation does not induce a significant improvement in postoperative cardiopulmonary function. Current studies are mostly limited to the assessment of pulmonary function recovery in the early postoperative phase; there are few reports on the recovery of pulmonary functions after long-term pulmonary rehabilitation exercise.

Our research team is conducting a prospective study on long-term postoperative pulmonary rehabilitation based on telemedicine platforms in 500 patients, including a pulmonary rehabilitation remote monitoring group, in which patients are followed up and managed using wearable devices with remote monitoring functions and rehabilitation management systems; and a pulmonary rehabilitation conventional management group, in which patients are followed up and managed using common means of social communication, including phone calls, text messages, and WeChat. In this ongoing study, we are applying digital technologies and multidisciplinary individualized comprehensive interventions to improve respiratory function, relieve postoperative symptoms, improve daily activity endurance, and promote wound healing after thoracic surgery. Remote wearable devices are used for the collection of physiological parameters and data analysis. We also plan to integrate internet technologies into postoperative monitoring and rehabilitation after thoracic surgery, in order to alleviate or control the complications of minimally invasive lung cancer surgery, and eliminate surgery-induced dysfunctions and psychological issues. In addition, we educate patients on how to improve exercise and activity endurance, improve self-care ability, and reduce the risk of hospitalization. The study is currently mid-stage, and preliminary data have confirmed the feasibility of telemedicine-based post-thoracic surgery rehabilitation. We believe that the results of this study can provide evidence to verify the necessity of postoperative rehabilitation in patients with lung cancer, promote the application of telemedicine techniques in the field of post-tumor surgery rehabilitation, facilitate the introduction and promotion of post-lung cancer surgery telerehabilitation programs in China, and inspire standardized procedures for post-lung cancer surgery telerehabilitation.

## Author Contributions

SX and GW provided the initial idea for this review. YF, MN and FW were in charge of data acquisition and drafting of the article. TD, WY and WJ revised the article. All authors read and approved the final manuscript.

## Funding

National Key Research and Development Program (2019YFE0105600).

## Conflict of Interest

The authors declare that the research was conducted in the absence of any commercial or financial relationships that could be construed as a potential conflict of interest.

## Publisher’s Note

All claims expressed in this article are solely those of the authors and do not necessarily represent those of their affiliated organizations, or those of the publisher, the editors and the reviewers. Any product that may be evaluated in this article, or claim that may be made by its manufacturer, is not guaranteed or endorsed by the publisher.

## References

[1] Bray F Ferlay J Soerjomataram I Siegel RL Torre L A Jemal A. Global Cancer Statistics 2018: GLOBOCAN Estimates of Incidence and Mortality Worldwide for 36 Cancers in 185 Countries. CA Cancer J Clin (2018) 68:394–424. doi: 10.3322/caac.21492 30207593

[2] Shiono S Abiko M Sato T. Postoperative Complications in Elderly Patients After Lung Cancer Surgery. Interact Cardiovasc Thorac Surg (2013) 16:819–23. doi: 10.1093/icvts/ivt034 PMC3653459 23427311

[3] Aliboni L Tullio M Pennati F Lomauro A Carrinola R Carrafiello G. Functional Analysis of the Airways After Pulmonary Lobectomy Through Computational Fluid Dynamics. Sci Rep (2022) 12:3321. doi: 10.1038/s41598-022-06852-x 35228582 PMC8885819

[4] Matsumoto R Takamori S Yokoyama S Hashiguchi T Murakami D Yoshiyama K. Lung Function in the Late Postoperative Phase and Influencing Factors in Patients Undergoing Pulmonary Lobectomy. J Thorac Dis (2018) 10:2916–23. doi: 10.21037/jtd.2018.05.27 PMC6006118 29997957

[5] Ueda K Tanaka T Hayashi M Li TS Kaneoka T Tanaka N. Compensation of Pulmonary Function After Upper Lobectomy Versus Lower Lobectomy. J Thorac Cardiovasc Surg (2011) 142:762–7. doi: 10.1016/j.jtcvs.2011.04.037 21679975

[6] Brunelli A Kim AW Berger K I Addrizzo-Harris DJ. Physiologic Evaluation of the Patient With Lung Cancer Being Considered for Resectional Surgery: Diagnosis and Management of Lung Cancer, 3rd Ed: American College of Chest Physicians Evidence-Based Clinical Practice Guidelines. Chest (2013) 143:e166S–90S. doi: 10.1378/chest.12-2395 23649437

[7] Zeiher BG Gross TJ Kern JA Lanza L A Peterson MW. Predicting Postoperative Pulmonary Function in Patients Undergoing Lung Resection. Chest (1995) 108:68–72. doi: 10.1378/chest.108.1.68 7606994

[8] Kang H J Lee SS. Comparison of Predicted Postoperative Lung Function in Pneumonectomy Using Computed Tomography and Lung Perfusion Scans. J Chest Surg (2021) 54:487–93. doi: 10.5090/jcs.21.084 PMC8646060 34815369

[9] Zhang R Lee SM Wigfield C Vigneswaran W T Ferguson MK. Lung Function Predicts Pulmonary Complications Regardless of the Surgical Approach. Ann Thorac Surg (2015) 99:1761–7. doi: 10.1016/j.athoracsur.2015.01.030 25818569

[10] Khullar OV Wei JW Wagh K BiNongo JN Pickens A Sancheti MS. Preoperative Lung Function Is Associated With Patient-Reported Outcomes After Lung Cancer Surgery. Ann Thorac Surg (2021) 112:415–22. doi: 10.1016/j.athoracsur.2020.09.016 33130117

[11] Kocher GJ Gioutsos KP Ahler M Funke-Chambour M Ott SR Dorn P. Perioperative Lung Function Monitoring for Anatomic Lung Resections. Ann Thorac Surg (2017) 104:1725–32. doi: 10.1016/j.athoracsur.2017.06.018 28964423

[12] Poghosyan H Sheldon LK Leveille S G Cooley ME. Health-Related Quality of Life After Surgical Treatment in Patients With Non-Small Cell Lung Cancer: A Systematic Review. Lung Cancer (2013) 81:11–26. doi: 10.1016/j.lungcan.2013.03.013 23562675

[13] Ozturk A Sarihan S Ercan I Karadag M. Evaluating Quality of Life and Pulmonary Function of Long-Term Survivors of Non-Small Cell Lung Cancer Treated With Radical or Postoperative Radiotherapy. Am J Clin Oncol (2009) 32:65–72. doi: 10.1097/COC.0b013e31817e6ec2 19194128

[14] Chang NW Lin KC Lee SC Chan JY Lee Y H Wang KY. Effects of an Early Postoperative Walking Exercise Programme on Health Status in Lung Cancer Patients Recovering From Lung Lobectomy. J Clin Nurs (2014) 23:3391–402. doi: 10.1111/jocn.12584 24646333

[15] Miserocchi G Beretta E Rivolta I. Respiratory Mechanics and Fluid Dynamics After Lung Resection Surgery. Thorac Surg Clin (2010) 20:345–57. doi: 10.1016/j.thorsurg.2010.03.001 20619225

[16] Brunelli A Xiume F Refai M Salati M Marasco R Sciarra V. Evaluation of Expiratory Volume, Diffusion Capacity, and Exercise Tolerance Following Major Lung Resection: A Prospective Follow-Up Analysis. Chest (2007) 131:141–7. doi: 10.1378/chest.06-1345 17218568

[17] Cukic V. Reduction of Pulmonary Function After Surgical Lung Resections of Different Volume. Med Arch (2014) 68:231–5. doi: 10.5455/medarh.2014.68.231-235 PMC4240564 25568542

[18] Shibazaki T Mori S Harada E Shigemori R Kato D Matsudaira H. Measured Versus Predicted Postoperative Pulmonary Function at Repeated Times Up to 1 Year After Lobectomy. Interact Cardiovasc Thorac Surg (2021) 33:727–33. doi: 10.1093/icvts/ivab168 PMC8691694 34115872

[19] Nezu K Kushibe K Tojo T Takahama M Kitamura S. Recovery and Limitation of Exercise Capacity After Lung Resection for Lung Cancer. Chest (1998) 113:1511–6. doi: 10.1378/chest.113.6.1511 9631786

[20] Yokoba M Ichikawa T Harada S Naito M Sato Y Katagiri M. Postoperative Pulmonary Function Changes According to the Resected Lobe: A 1-Year Follow-Up Study of Lobectomized Patients. J Thorac Dis (2018) 10:6891–902. doi: 10.21037/jtd.2018.11.108 PMC6344756 30746235

[21] Liu M Wampfler JA Dai J Gupta R Xue Z Stoddard SM. Chest Wall Resection for Non-Small Cell Lung Cancer: A Case-Matched Study of Postoperative Pulmonary Function and Quality of Life. Lung Cancer (2017) 106:37–41. doi: 10.1016/j.lungcan.2017.01.014 28285692

[22] Cesario A Ferri L Galetta D Pasqua F Bonassi S Clini E. Post-Operative Respiratory Rehabilitation After Lung Resection for Non-Small Cell Lung Cancer. Lung Cancer (2007) 57:175–80. doi: 10.1016/j.lungcan.2007.02.017 17442449

[23] Saito H Shiraishi A Nomori H Matsui H Yoshida K Matsue Y. Impact of Age on the Recovery of Six-Minute Walking Distance After Lung Cancer Surgery: A Retrospective Cohort Study. Gen Thorac Cardiovasc Surg (2020) 68:150–7. doi: 10.1007/s11748-019-01191-7 31485843

[24] Do JG Park CH Lee Y T Yoon KJ. Association Between Underweight and Pulmonary Function in 282,135 Healthy Adults: A Cross-Sectional Study in Korean Population. Sci Rep (2019) 9:14308. doi: 10.1038/s41598-019-50488-3 31586079 PMC6778122

[25] Cho S J Stout-Delgado HW. Aging and Lung Disease. Annu Rev Physiol (2020) 82:433–59. doi: 10.1146/annurev-physiol-021119-034610 PMC7998901 31730381

[26] Mori S Shibazaki T Noda Y Kato D Nakada T Asano H. Recovery of Pulmonary Function After Lung Wedge Resection. J Thorac Dis (2019) 11:3738–45. doi: 10.21037/jtd.2019.09.32 PMC6790438 31656646

[27] Mimae T Miyata Y Kumada T Handa Y Tsutani Y Okada M. Interstitial Pneumonia and Advanced Age Negatively Influence Postoperative Pulmonary Function. Interact Cardiovasc Thorac Surg (2022) 34(5):753–59. doi: 10.1093/icvts/ivac014 PMC9070519 35137092

[28] Ferreira V Lawson C Ekmekjian T Carli F Scheede-Bergdahl C Chevalier S. Effects of Preoperative Nutrition and Multimodal Prehabilitation on Functional Capacity and Postoperative Complications in Surgical Lung Cancer Patients: A Systematic Review. Support Care Cancer (2021) 29:5597–610. doi: 10.1007/s00520-021-06161-5 33768372

[29] Pan J Xu L Lam TH Jiang CQ Zhang WS Jin YL. Association of Adiposity With Pulmonary Function in Older Chinese: Guangzhou Biobank Cohort Study. Respir Med (2017) 132:102–8. doi: 10.1016/j.rmed.2017.10.003 29229080

[30] Fukui M Suzuki K Matsunaga T Oh S Takamochi K. Importance of Smoking Cessation on Surgical Outcome in Primary Lung Cancer. Ann Thorac Surg (2019) 107:1005–9. doi: 10.1016/j.athoracsur.2018.12.002 30610851

[31] Lee PN Forey BA Thornton A J Coombs KJ. The Relationship of Cigarette Smoking in Japan to Lung Cancer, COPD, Ischemic Heart Disease and Stroke: A Systematic Review. F1000Res (2018) 7:204. doi: 10.12688/f1000research.14002.1 30800285 PMC6367657

[32] Harada H Okada M Sakamoto T Matsuoka H Tsubota N. Functional Advantage After Radical Segmentectomy Versus Lobectomy for Lung Cancer. Ann Thorac Surg (2005) 80:2041–5. doi: 10.1016/j.athoracsur.2005.06.010 16305841

[33] Macke RA Schuchert MJ Odell DD Wilson DO Luketich J D Landreneau RJ. Parenchymal Preserving Anatomic Resections Result in Less Pulmonary Function Loss in Patients With Stage I Non-Small Cell Lung Cancer. J Cardiothorac Surg (2015) 10:49. doi: 10.1186/s13019-015-0253-6 25888465 PMC4382835

[34] Saito H Nakagawa T Ito M Imai K Ono T Minamiya Y. Pulmonary Function After Lobectomy Versus Segmentectomy in Patients With Stage I Non-Small Cell Lung Cancer. World J Surg (2014) 38:2025–31. doi: 10.1007/s00268-014-2521-3 24682312

[35] Keenan RJ Landreneau RJ Maley RH Jr. Singh D Macherey R Bartley S. Segmental Resection Spares Pulmonary Function in Patients With Stage I Lung Cancer. Ann Thorac Surg (2004) 78:228–33. doi: 10.1016/j.athoracsur.2004.01.024 15223434

[36] Rationale and Design of The National Emphysema Treatment Trial: A Prospective Randomized Trial of Lung Volume Reduction Surgery. The National Emphysema Treatment Trial Research Group. Chest (1999) 116:1750–61. doi: 10.1378/chest.116.6.1750 10593802

[37] Seok Y Jheon S Cho S. Serial Changes in Pulmonary Function After Video-Assisted Thoracic Surgery Lobectomy in Lung Cancer Patients. Thorac Cardiovasc Surg (2014) 62:133–9. doi: 10.1055/s-0033-1343980 23619593

[38] Wei S Chen F Liu R Fu D Wang Y Zhang B. Outcomes of Lobectomy on Pulmonary Function for Early Stage Non-Small Cell Lung Cancer (NSCLC) Patients With Chronic Obstructive Pulmonary Disease (COPD). Thorac Cancer (2020) 11:1784–9. doi: 10.1111/1759-7714.13445 PMC7592038 32374491

[39] Baldi S Ruffini E Harari S Roviaro GC Nosotti M Bellaviti N. Does Lobectomy for Lung Cancer in Patients With Chronic Obstructive Pulmonary Disease Affect Lung Function? A Multicenter National Study. J Thorac Cardiovasc Surg (2005) 130:1616–22. doi: 10.1016/j.jtcvs.2005.06.049 16308007

[40] Brown LM Rannels S R Rannels DE. Implications of Post-Pneumonectomy Compensatory Lung Growth in Pulmonary Physiology and Disease. Respir Res (2001) 2:340–7. doi: 10.1186/rr84 PMC64801 11737933

[41] Fisher J M Simnett JD. Morphogenetic and Proliferative Changes in the Regenerating Lung of the Rat. Anat Rec (1973) 176:389–95. doi: 10.1002/ar.1091760403 4723402

[42] Hsia CCW. Quantitative Morphology of Compensatory Lung Growth. Eur Respir Rev (2006) 15:148–56. doi: 10.1183/09059180.00010105

[43] Sengul AT Sahin B Celenk C Basoglu A. Postoperative Lung Volume Change Depending on the Resected Lobe. Thorac Cardiovasc Surg (2013) 61:131–7. doi: 10.1055/s-0032-1322625 23475808

[44] Nomori H Shiraishi A Cong Y Shoji K Misawa M Sugimura H. Impact of Induction Chemoradiotherapy on Pulmonary Function After Lobectomy for Lung Cancer. J Thorac Cardiovasc Surg (2018) 155:2129–2137 e1. doi: 10.1016/j.jtcvs.2017.12.081 29395208

[45] Abratt R P Morgan GW. Lung Toxicity Following Chest Irradiation in Patients With Lung Cancer. Lung Cancer (2002) 35:103–9. doi: 10.1016/s0169-5002(01)00334-8 11804681

[46] Shin S Choi YS Jung JJ Im Y Shin SH Kang D. Impact of Diffusing Lung Capacity Before and After Neoadjuvant Concurrent Chemoradiation on Postoperative Pulmonary Complications Among Patients With Stage IIIA/N2 Non-Small-Cell Lung Cancer. Respir Res (2020) 21(1):13. doi: 10.1186/s12931-019-1254-0 31924201 PMC6954564

[47] Zhu Y Li JQ Chang Q Qiang HP Lu JH Feng H. Impact of Neoadjuvant Immunotherapy on Pulmonary Function and Perioperative Outcomes in Patients with Resectable Non-Small Cell Lung Cancer. Zhonghua Yi Xue Za Zhi (2022) 102:393–8. doi: 10.3760/cma.j.cn112137-20211009-02226 35144337

[48] Brunelli A Salati M. Preoperative Evaluation of Lung Cancer: Predicting the Impact of Surgery on Physiology and Quality of Life. Curr Opin Pulm Med (2008) 14:275–81. doi: 10.1097/MCP.0b013e328300caac 18520258

[49] Jones LW Peddle CJ Eves ND Haykowsky MJ Courneya KS Mackey JR. Effects of Presurgical Exercise Training on Cardiorespiratory Fitness Among Patients Undergoing Thoracic Surgery for Malignant Lung Lesions. Cancer (2007) 110:590–8. doi: 10.1002/cncr.22830 17582629

[50] Fiorelli A Morgillo F Milione R Pace MC Passavanti MB Laperuta P. Control of Post-Thoracotomy Pain by Transcutaneous Electrical Nerve Stimulation: Effect on Serum Cytokine Levels, Visual Analogue Scale, Pulmonary Function and Medication. Eur J Cardiothorac Surg (2012) 41:861–8. doi: 10.1093/ejcts/ezr108 22219414

[51] Messaggi-Sartor M Marco E Martinez-Tellez E Rodriguez-Fuster A Palomares C Chiarella S. Combined Aerobic Exercise and High-Intensity Respiratory Muscle Training in Patients Surgically Treated for Non-Small Cell Lung Cancer: A Pilot Randomized Clinical Trial. Eur J Phys Rehabil Med (2019) 55:113–22. doi: 10.23736/S1973-9087.18.05156-0 29984565

[52] Solberg Nes L Liu H Patten CA Rausch SM Sloan JA Garces YI. Physical Activity Level and Quality of Life in Long Term Lung Cancer Survivors. Lung Cancer (2012) 77:611–6. doi: 10.1016/j.lungcan.2012.05.096 PMC3882512 22681871

[53] Vagvolgyi A Rozgonyi Z Kerti M Agathou G Vadasz P Varga J. Effectiveness of Pulmonary Rehabilitation and Correlations in Between Functional Parameters, Extent of Thoracic Surgery and Severity of Post-Operative Complications: Randomized Clinical Trial. J Thorac Dis (2018) 10:3519–31. doi: 10.21037/jtd.2018.05.202 PMC6051851 30069349

[54] Zhou T Sun C. Effect of Physical Manipulation Pulmonary Rehabilitation on Lung Cancer Patients After Thoracoscopic Lobectomy. Thorac Cancer (2022) 13:308–15. doi: 10.1111/1759-7714.14225 PMC8807280 34882313

[55] Zou H Qin Y Gong F Liu J Zhang J Zhang LJGN. ABCDEF Pulmonary Rehabilitation Program can Improve the Mid-Term Lung Function of Lung Cancer Patients After Thoracoscopic Surgery: A Randomized Controlled Study. Geriatr Nurs (2022) 44:76–83. doi: 10.1016/j.gerinurse.2021.12.021 35074540

[56] Choi J Yang Z Lee J Lee JH Kim HK Yong HS. Usefulness of Pulmonary Rehabilitation in Non-Small Cell Lung Cancer Patients Based on Pulmonary Function Tests and Muscle Analysis Using Computed Tomography Images. Cancer Res Treat (2021). doi: 10.4143/crt.2021.769 PMC9296947 34696566

[57] Edvardsen E Skjonsberg OH Holme I Nordsletten L Borchsenius F Anderssen SA. High-Intensity Training Following Lung Cancer Surgery: A Randomised Controlled Trial. Thorax (2015) 70:244–50. doi: 10.1136/thoraxjnl-2014-205944 25323620

[58] Jonsson M Ahlsson A Hurtig-Wennlof A Vidlund M Cao Y Westerdahl E. In-Hospital Physiotherapy and Physical Recovery 3 Months After Lung Cancer Surgery: A Randomized Controlled Trial. Integr Cancer Ther (2019) 18:1534735419876346. doi: 10.1177/1534735419876346 31530046 PMC6751530

[59] Cavalheri V Jenkins S Cecins N Gain K Phillips MJ Sanders LH. Exercise Training for People Following Curative Intent Treatment for Non-Small Cell Lung Cancer: A Randomized Controlled Trial. Braz J Phys Ther (2017) 21:58–68. doi: 10.1016/j.bjpt.2016.12.005 28442076 PMC5537441

